# Nuclear Structure—The Future With Radioactive Beams

**DOI:** 10.6028/jres.105.005

**Published:** 2000-02-01

**Authors:** D. D. Warner

**Affiliations:** CLRC Daresbury Laboratory, Daresbury, Warrington, WA4 4AD, UK

**Keywords:** neutron-proton pairing, nuclear structure, radioactive beams

## Abstract

Some examples of new phenomena expected at extreme values of neutron and proton number are discussed, including soft dipole modes and neutron-proton pairing. First results on the *N* = *Z* nucleus ^62^Ga are presented in the context of the competition between *T* = 0 and 1 states in odd-odd, self-conjugate nuclei.

## 1. Introduction

The nucleus represents a unique many-body problem involving a large, but finite, number of strongly interacting particles. The most fundamental approach to nuclear structure is represented by self-consistent, many-body theory whose goal is the definition of an effective interaction which describes the nucleon-nucleon force inside the nuclear medium. This effective interaction is represented in terms of the nuclear mean field formed by the average of all the interactions in the nucleus and its validity can be probed by looking at nuclei under extreme conditions.

In looking for the new frontiers, it seems clear that the usual expedient of going to higher energies is no longer valid; we already have access to high enough energy to reach the Fermi energy and beyond and to attain maximum angular momentum. The frontier now lies at the extremes of nuclear existence where we can amplify the sensitivity of our mean field predictions by going to extreme values of *N*/*Z* to verify the density dependence of the effective interaction. To reach these exotic regions of the nuclear chart and obtain the necessary experimental information requires the use of radioactive beams at Coulomb barrier energies and beyond.

Some examples of the basic precepts and predictions upon which our current understanding of nuclear structure is based are listed below.

### 1.1 Limits of Existence

There are three natural limits of the nuclear chart.

- The neutron drip line is known only up to about mass 30, after which its predicted position lies progressively further away from the last experimentally studied nucleus. Existing many body predictions of its location vary considerably [1] once the region of known binding energies is left behind. In addition, the development of an increasing neutron excess can manifest itself in new collective modes and this will be the primary example dealt with here. The neutron drip line is the line that defines the margin of the chart of nuclides, beyond which, nuclei can undergo decay by emitting a single nucleon.
- The region of the proton drip line is, by comparison, much better known but structure and phenomena there are only just beginning to be studied. Examples are p-decay where a proton is caught between the top of the nuclear well and the Coulomb potential, the possibility of p-haloes, and the structure of heavy *N* = *Z* nuclei.
- The existence and location of the superheavy elements. There is a long-standing prediction of spherical shell closure at *N* = 184, *Z* = 114 and a more recent indication that the proton shell may occur at *Z* = 126 [2]. The main question here is to what extent use of n-rich beams can enhance cross section and how intense they can be made. This requires reaction studies on the fusion of n-rich systems.

### 1.2 Magic Numbers—Are They Universal?

Some recent calculations [3] indicate a gradual erosion of well-established shell gaps as the neutron drip line is approached in various mass regions. The effect is qualitatively equivalent to a reduction of the *l.s.* and *l*^2^ forces so that the shell structure tends back towards that of the harmonic oscillator. The consequent absence of the high-*j* intruder orbital from each shell would give rise to significant changes in collective structure and also a much improved applicability of SU(3) symmetry.

### 1.3 Role of n-p Symmetry

Access to the heavier *N* = *Z* nuclei allows the role of isospin in nuclear structure to be explored for the first time and, in particular, the part played by n-p pairing. This will be discussed in more detail later.

Only a limited and personal view of some of the most important questions to be answered with radioactive beams have been mentioned so far. One could make a much longer and more complete list which included the massive contribution RNBs will make to astrophysics, for example. However, the remainder of this paper will focus on a more detailed examination of a few specific highlights.

## 2. The Neutron Skin

In recent years, experiments with radioactive beams from projectile fragmentation facilities have revealed [4] the presence of a neutron halo in several of the lightest nuclei on the neutron drip line. This is now understood to arise when the last one or two neutrons are in low angular momentum orbits very near the top of the well so that their wave functions have a very extended distribution which is manifest empirically in an anomalously large matter radius. There is, however, a distinctly different phenomenon which is predicted in some Hartree–Fock calculations [5, 6], to occur in heavier nuclei in which an excess of several neutrons builds up so that the neutron density actually extends out significantly further than that of the protons, resulting in a mantle of dominantly neutron matter.

**Fig. 1:**
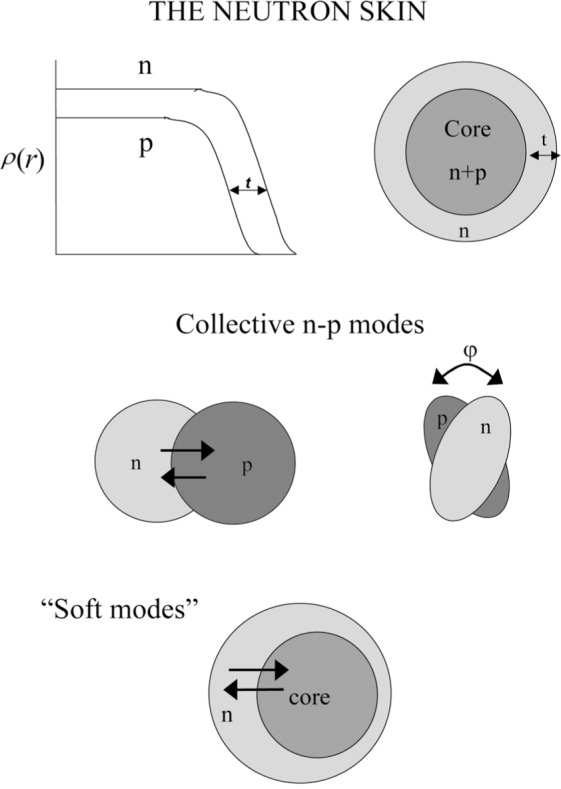
Schematic illustration of the neutron skin and collective dipole modes.

This situation is illustrated schematically in Fig. 1. The presence of this neutron “skin” may affect collective modes of nuclear excitation which involve the out-of-phase motion of neutrons against protons, such as the giant dipole resonance (GDR) [7] and the scissors mode [8]. There is also then the possibility of a “soft” dipole mode [9] in which the core nucleons move against the more weakly bound skin neutrons.

In the normal application of the Interacting Boson Model (IBM) [10] to nuclei nearer to stability, the nucleon pairs can be described microscopically in terms of the known shell structure. In the problem addressed here, the shell structure at the extremes of stability is not known and no such link can be established quantitatively at this stage. Nevertheless, it has recently been demonstrated [11] that the associated algebra of U(6) can be used to provide an elegant and simple way of looking at the quadrupole degrees of freedom available to the three component system which results from the development a neutron skin sufficient in extent to be at least partially decoupled from the core nucleons.

The starting point is taken as a triple product involving an additional algebra $U_{v_{s}}(6)$, describing the skin, with the remaining core neutrons being described by $U_{v_{c}}(6)$. The dynamical algebra of the system is then

$$\begin{array}{ccccc} U_{\pi }(6) & ⊗ & U_{v_{c}}(6) & ⊗ & U_{v_{s}}(6)\\ ↓ & & ↓ & & ↓\\ \left[N_{\pi }\right] & & \left[N_{v_{c}}\right] & & \left[N_{v_{s}}\right] \end{array},$$ (1)

where each U(6) algebra is characterised by a number of bosons *N_i_* that are coupled symmetrically to [*N_i_*].

The fact that the skin neutrons are assumed to interact weakly with the core neutrons and protons, which interact strongly with each other, is represented in the reduction of Eq. (1) by coupling the corresponding U(6) algebra of the neutron skin *after* those describing the core nucleons. The reduction thus proceeds as

$$\begin{array}{c} U_{\pi }(6)⊗U_{v_{c}}(6)⊗U_{v_{s}}(6)\\ ⊃U_{\pi v_{c}}(6)⊗U_{v_{s}}(6)⊃U_{\pi v_{c}v_{s}}(6). \end{array}$$ (2)

The triple-sum algebra $U_{\pi v_{c}v_{s}}(6)$ has a subalgebra structure familiar from IBM-1, and, specifically, the three usual limits [10], U(5), SU(3) and O(6), can be obtained as subchains of Eq. (2). However, the results derived below are not only valid in the dynamical symmetries but also for intermediate situations. The presence of $U_{\pi v_{c}v_{s}}(6)$ would then imply that an appropriate identical mixture of U(5), SU(3) and O(6) describes all three subsystems *π*, *v*_c_ and *v*_s_ and hence that all three have the same deformation.

It is the coupled nature of the algebra U*_πv_*(6) in IBM-2 that permits states with mixed symmetry [12]. In the reduction Eq. (2) $U_{\pi v_{c}}(6)$ is characterised by irreducible representations [*N*_c_ − *f*, *f*] where *N*_c_ is the number of nucleon pairs in the core, $N_{c}=N_{\pi }+N_{v_{c}}$. The lowest states are then contained in the representation [*N*_c_, 0], which denotes the totally symmetric coupling. The lowest states of mixed symmetry are in the next representation, [*N*_c_ − 1, 1]. The triple-sum algebra $U_{\pi v_{c}v_{s}}(6)$ is characterised by up to three rows, with the lowest couplings arising from $\left[N_{c},0]\times [N_{v_{s}}\right]$ being [*N*, 0, 0] and [*N* − 1, 1, 0], *N* denoting the total number of bosons. Hence the first non-symmetric representation resulting from the triple-sum algebra describes *symmetric* coupling of the core nucleons and *non-symmetric* coupling of the skin neutrons. However, the non-symmetric representation [*N* − 1, 1, 0] of $U_{\pi v_{c}v_{s}}(6)$ may also arise from the product $\left[N_{c}-1,1]\times [N_{v_{s}}\right]$. In this case, it is the core nucleons which are coupled non-symmetrically. The result is that there are now *two* non-symmetric modes, one representing out-of-phase motion between the neutrons and protons in the core and the other denoting an oscillation between the core and the skin where the core protons carry the core neutrons with them.

The relative energies of the soft and normal mixed symmetry modes can now be estimated by considering the Hamiltonian

$$\hat{H}=\alpha {\hat{C}}_{2}[U_{\pi v_{c}}(6)]+\beta {\hat{C}}_{2}[U_{\pi v_{c}v_{s}}(6)],$$ (3)

where *Ĉ*_2_[U*_ij_* (6)] denotes the quadratic Casimir operator of the algebra U*_ij_* (6) obtained by adding the generators of subsystems *i* and *j*. The energy of the normal mode is then $2\alpha (N_{\pi }+N_{v_{c}})+2\beta N$ while for the soft mode it is 2*βN*. Considering now the specific case of SU(3), the mixed symmetry mode corresponds to the scissors excitation of Fig. 1, where the centre of gravity of the orbital M1 strength distribution in rare earth nuclei indicates an excitation energy for the normal mode of *E_x_* ~ 3 MeV. The signs of *α* and *β* in Eq. (3) are determined to be positive by the requirement that the fully symmetric representation of U(6) should lie lowest in energy. Assuming, in addition, that these two constants have roughly equal magnitude, then if the number of neutrons in the skin is small compared to the total, the soft mode should appear at approximately half the energy of the normal one, resulting in a predicted energy for the soft mode of *E_x_* ~ 1.5 MeV.

In even-even nuclei the existence of 1^+^ scissors states excited in (*e*, *e*′) or (*γ*, *γ*′) is by now well established. The predicted M1 strength towards the 1^+^ state is

$$B(M1;0_{G}^{+}\to 1_{S}^{+})=\frac{3}{4\pi }\;{\left(g_{\pi }-g_{v}\right)}^{2}\;f(N)N_{\pi }N_{v},$$ (4)

where *g_π_* and *g_v_* are the boson *g* factors. The function *f* (*N*) is known analytically in the three limits of the IBM [12].

A corresponding expression can be derived for the dipole strength to the soft scissors state by considering the separate contributions to the M1 operator from the core and the skin neutrons,

$$\hat{T}(M1)=g_{\pi }{\hat{L}}_{\pi }+g_{v}{\hat{L}}_{v}=g_{\pi }{\hat{L}}_{\pi }+g_{v}{\hat{L}}_{v_{c}}+g_{v}{\hat{L}}_{v_{s}},$$ (5)

and this yields

$$B(M1;0_{G}^{+}\to 1_{\mathrm{SS}}^{+})=\frac{3}{4\pi }\;{\left(g_{\pi }-g_{v}\right)}^{2}\;f(N)\frac{N_{\pi }^{2}N_{v_{s}}}{N_{\pi }+N_{v_{c}}}.$$ (6)

From Eqs. (4) and (6) one finds the following simple result for the ratio of *B*(M1)’s in the soft and normal scissors modes:

$$\frac{B(M1;0_{G}^{+}\to 1_{\mathrm{SS}}^{+})}{B(M1;0_{G}^{+}\to 1_{S}^{+})}=\frac{N_{\pi }N_{v_{s}}}{(N_{\pi }+N_{v_{c}})N_{v}}.$$ (7)

This value is identical to that found previously for the E1 sum rule ratio of the soft and giant dipole resonances [13]; the result depends only on the relative number of constituents in the subsystems, rather than on the specific algebra chosen.

Thus, evidence for enhanced M1 strength at low excitation energies could represent a signature for the onset of a neutron skin and hence the approach of the neutron drip line. The experimental technique most likely to reveal such features would be relativistic Coulomb excitation using radioactive beams from a projectile fragmentation facility. In Coulex experiments at barrier energies, the M1 excitation probability is severely hindered relative to the *E*2 because of a (*υ*/*c*)^2^ dependence of the cross section in the former case. At facilities such as GSI, *υ*/*c* ≃ 0.5 is possible, rendering the consequent reduction in the M1 cross section manageable.

## 3. The *N* = *Z* Line

Above ^56^Ni, the locus of *N* = *Z* nuclei rapidly approaches the proton drip line, eventually crossing it just above ^100^Sn. The medium heavy, *N* = *Z* nuclei in this *pfg*_9/2_ shell are of crucial importance in nuclear structure for two principal reasons: they have neutrons and protons in the same valence shell and the 28–50 shell is large enough for the nuclei to exhibit all aspects of nuclear collective behaviour. These characteristics result in a number of features unique to this class of nuclei, resulting from the role played by the neutron-proton exchange symmetry in the collective structure in this region. Examples include the study of mirror nuclei at high spin, the likely magnitude reached by isospin admixtures with increasing *Z* and, perhaps most intriguing of all, the role played by neutron-proton pairing.

The new ingredient that appears on the *N* = *Z* line is the symmetry associated with the isospin quantum number, *T*, and again, an algebraic approach can be adopted for the *N* = *Z* nuclei. There are two isospin invariant versions of the IBM; IBM-3 [14] with *T* = 1 and *M_T_* = 0, ±1 and IBM-4 [15] which incorporates a fourth kind of boson corresponding to the *T* = 0 neutron-proton pair. In IBM-4 the *T* = 1 bosons are assigned an intrinsic spin *S* = 0 and complemented with a set of *T* = 0, *S* = 1 bosons. The rationale behind this choice is that the two-particle isospin-spin combinations (*TS*) = (10) and (01) are the ones favoured by Wigner’s SU(4) classification [16] which is known to have physical significance in light nuclei. In heavier nuclei SU(4) symmetry is increasingly broken but IBM-4 may remain a valid approximation (as it does in *sd*-shell nuclei [17, 18] if the boson *L* and *S* are equated to the *pseudo* orbital and spin angular momentum quantum numbers of two fermions [19].

Recently it has been shown possible to establish a connection between IBM-3 and IBM-4 [20]. This was done by constructing a classification in IBM-4 that does not conserve SU(4) symmetry and which has a subset of states in direct correspondence with IBM-3 states. Moreover, this link between IBM-3 and IBM-4 provided an ideal tool to study the competition between *T* = 0 and *T* = 1 pairing.

The dynamical algebra of IBM-4 is U(36) since it involves the same orbital degrees of freedom coupled with an additional six isospin-spin [(*TS*) = (01) and (10)] degrees of freedom. The usual IBM-4 classification, as proposed by Elliott and Evans [15] is

$$\begin{array}{l} \begin{array}{ccccccc} U(36) & ⊃ & \left(U_{L}(6\right) & ⊃ & \ldots & ⊃ & O_{L}(3))\\ ↓ & & ↓ & & & & ↓\\ \left[N\right] & & \left[N_{1}\ldots N_{6}\right] & & & & L \end{array},\\ \;\begin{array}{cccccc} ⊗ & \left(U_{TS}(6\right) & ⊃ & {\mathrm{SU}}_{TS}(4) & ⊃ & {\mathrm{SU}}_{T}(2)\\ & \;↓ & & ↓ & & ↓\\ & \left[N_{1}\ldots N_{6}\right] & & \left(\lambda \mu v\right) & & T \end{array}\\ \;\begin{array}{cc} ⊗ & {\mathrm{SU}}_{S}(2))\\ & \;↓\\ & \;S \end{array} \end{array}$$ (8)

where the orbital angular momentum *L* must be coupled with spin *S* to total angular momentum *J* (not shown). The novel element with respect to IBM-3 is the appearance of spin algebras, notably of Wigner’s SU*_TS_* (4).

The states of IBM-3 are a subset of those in IBM-4. The precise relationship cannot be established via the classification (8) but rather via one in which the isospin-spin reduction is replaced by

$$\begin{array}{cccccccc} (U_{TS}(6)⊃ & {\mathrm{SU}}_{T}(3) & ⊗ & {\mathrm{SU}}_{S}(3) & ⊃ & {\mathrm{SU}}_{T}(2) & ⊗ & {\mathrm{SU}}_{S}(2)\\ ↓ & ↓ & & ↓ & & ↓ & & ↓\\ \left[N_{1}\ldots N_{6}\right] & \left(\lambda_{T}\mu_{T}\right) & & \left(\lambda_{S}\mu_{S}\right) & & T & & S \end{array}$$ (9)

Those IBM-4 states which have an analogue in IBM-3 can now be identified: they are those which are scalar in SU*_S_*(3). This implies that adding an interaction which removes all non-scalar SU*_S_*(3) states from the low-energy spectrum will essentially reduce IBM-4 to IBM-3. The simplest example of such an interaction is the quadratic Casimir operator of SU*_S_*(3), *C*_2_[SU*_S_*(3)]. The physical relevance of an interaction of this type is that it determines the relative energy of the *T* = 0 and *T* = 1 collective pairs.

The effect of the *C*_2_[SU*_S_* (3)] operator can best be illustrated in odd-odd self-conjugate nuclei where the balance between *T* = 0 and *T* = 1 pairing is thought to be most crucial.

The effect of the *C*_2_[SU*_S_* (3)] operator can be calculated by considering the Hamiltonian

$$\hat{H}=aC_{2}[{\mathrm{SU}}_{TS}(4)]+bC_{2}[{\mathrm{SU}}_{S}(3)].$$ (10)

**Fig. 2:**
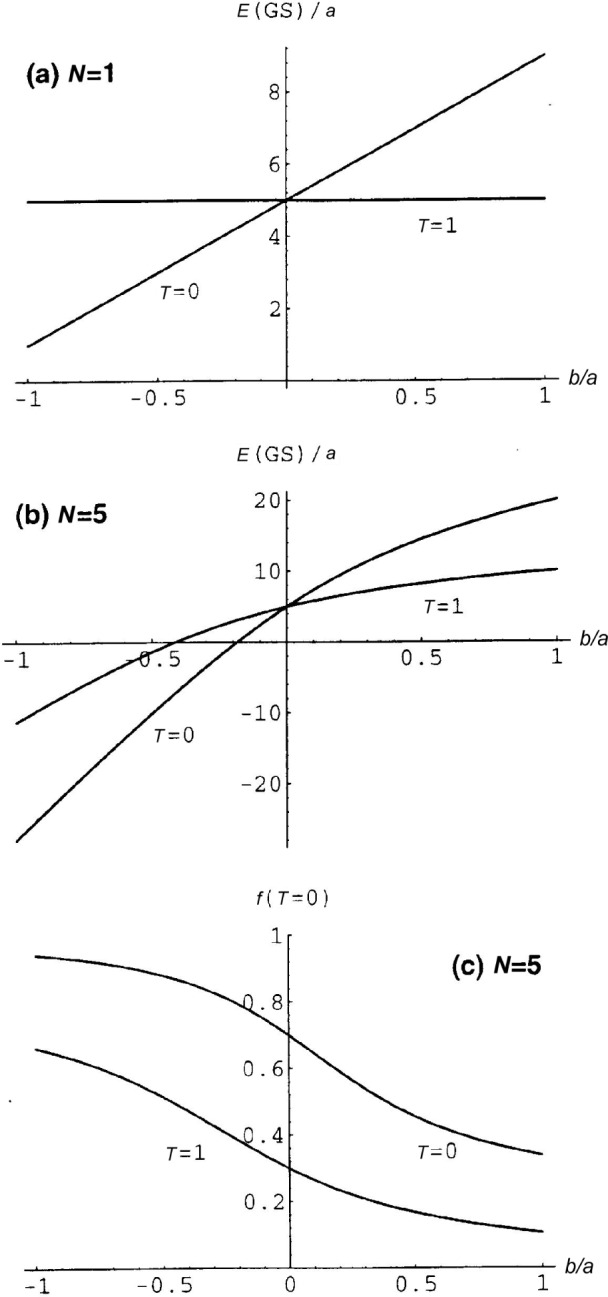
(a) Energy (in units *a*) of the lowest *T* = 0 and *T* = 1 eigenstates as a function of the ratio *b*/*a* of coefficients in Eq. (10) for *N* = 1 boson. (b) Same for *N* = 5 bosons. (c) The pair structure $f(T=0)\equiv \langle \hat{N}(T=0)\rangle /N$ as a function of *b*/*a* for the lowest *T* = 0 and *T* = 1 eigenstates of Eq. (10) for *N* = 5 bosons.

The results of diagonalising Eq. (10) are shown in Fig. 2(a–c). Quantities are plotted as a function of the ratio *b*/*a* of coefficients in Eq. (10) with *b*/*a* = 0 corresponding to the SU(4) limit. Figure 1(a) shows the *T* = 0 and *T* = 1 single boson energies. The expectation value of *C*_2_[SU*_S_* (3)] is zero for *S* = 0 and hence the *T* = 1 boson energy is unaffected by the chosen symmetry breaking term, while the energy of the *T* = 0 boson, with *S* = 1, has a linear dependence on *b*/*a*. Thus a shift between the energies of the *T* = 0 and *T* = 1 correlated pair states has been introduced. In Fig. 1(b) the effect of this shift on the lowest *T* = 0 and *T* = 1 states in an odd-odd nucleus with *N* = 5 is illustrated. The ground-state configuration changes at *b*/*a* = 0. The corresponding pair structure of the two states is shown in Fig. 1(c) in terms of the quantity *f* (*T* = 0) which represents the number of *T* = 0 bosons in the state as a fraction of the total boson number *N*, $f(T=0)\equiv \langle \hat{N}(T=0)\rangle /N$. The change in ground state which occurs at *b*/*a* = 0 is accompanied by a discontinuous change in *f* (*T* = 0), which jumps from (3*N* − 3)/8*N* to (5*N* + 3)/8*N*.

The pair structure of the ground state of *N* = *Z* nuclei depends on the relative energies of the *T* = 0 and *T* = 1 collective pairs; if one mode is greatly favoured, an almost pure pair structure, in terms of the isospin of the pairs, results. More realistically, if the two basic modes compete and have comparable energy, the resulting pair structure is a mixture of *T* = 0 and *T* = 1. In the particular case of degeneracy, corresponding to SU(4) symmetry, the ground state of *N* = *Z* nuclei contains 50 % of each pair type. This result differs from that of the earlier HFB treatments [21] where problems arise due to the lack of isospin invariance in this framework. In particular, in the HFB solutions for light nuclei, only one pairing mode dominates in the ground state, either *T* = 0 or *T* = 1, *T_z_* = ±1. More recent studies [20, 22, 23] have demonstrated that mixing between *T* = 0 and *T* = 1 modes and between *T_z_* = ±1 and *T_z_* = 0, *T* = 1 modes is unavoidable in an isospin invariant approach.

### 3.1 Experimental Signatures

It is clear from the above discussion that the study of odd-odd nuclei with *N* = *Z* may provide the best opportunity to observe the effects of the competition between *T* = 0 and *T* = 1 pairing correlations. The most obvious signature of the presence of n-p pairing will be the development of pairing gaps in the *T* = 0 and 1 states in the odd-odd system. Indeed, for the *T* = 1 states, the existence of such a gap follows naturally if the even-even isobaric analogue nucleus is sufficiently collective to exhibit one. For the *T* = 0 states, it is difficult to test this feature using reactions which favour only the yrast states. Nevertheless, the effect of the last neutron and proton forming part of a pair condensate should be quite striking. Recall that in an odd-odd nucleus with no n-p pairing, the rotational bands are characterised by *K* = |Ω_n_ ± Ω_p_|. Ω_n_ and Ω_p_ represent the projection of the single-particle angular momenta of the “unpaired” neutron and proton on the deformation axis. The bands are spaced simply according to the summed single-quasi-particle energies of the possible combinations of orbits with Ω_n_, Ω_p_. For example, the relevant energies of the orbits in the recently studied [24] ^74^Rb in the standard Nilsson model are shown in Table 1 for a prolate deformation *β*_2_ = 0.3 and would give rise to a total of 36 rotational band heads of each isospin below 2 MeV in ^74^Rb.

An additional signature could arise in *β*-decay. The simple isospin invariant IBM treatment cited earlier can be used to look at Gamow-Teller decay from *T* = 0 (1^+^) to *T* = 1(0^+^) states. The results imply a strong enhancement of the GT matrix element with boson number, consistent with the idea that the presence of n-p correlations can lead to a collective coherence in the *β*-decay. However, these results assume the SU(4) generator for the GT operator; while it is clear that this will no longer be applicable in the *N* = *Z* = 28–50 shell, recent use of the pseudo-spin concept has raised the possibility of a pseudo-spin symmetry in this shell which can still give rise to a degree of enhancement, albeit reduced [25]. In practice, testing of this concept involves study of the decay of the *T_Z_* = −1 even-even nucleus to the *T* = 0 odd-odd, since the ground state of the latter is invariably *T* = 1 in the region of interest.

A third signature could come from the study of band crossing phenomena where, if isospin is ignored, the coincidence of neutron and proton Fermi surfaces would give rise to simultaneous neutron and proton alignments. It has been pointed out that inclusion of both *T* = 0 and 1 pairing can delay one half of the alignment, which can no longer be attributed to neutrons or protons alone.

## 4. The *N* = Z Nucleus G3162a

In contrast to the situation in the *sd* shell, odd-odd *N* = *Z* systems heavier than ^40^Ca have *T* = 1 ground states, the only known exception being ^58^Cu. Above ^58^Cu, little or no information on excited states exists, with the exception of the recently studied case of ^74^Rb [74]. However, a recent study [27] resulted in the first experimental information on the decay scheme of ^62^Ga and, most importantly, identified the lowest *T* = 0 states.

The near yrast states in ^62^Ga were populated using the reaction ^40^Ca(^24^Mg, pn)^62^Ga at a beam energy of 65 MeV provided by the ATLAS facility at Argonne National Laboratory. An initial experiment used a self-supporting, unbacked target of thickness 500 μg/cm^2^. This was thin enough for the recoils to enter the Argonne Fragment Mass Analyser [28] which was coupled to a split anode ionization chamber, with the aim of obtaining isotopically pure identification spectra. Gamma rays were measured using the AYEBALL gamma-ray spectrometer [29], which consisted of five 70 % relative efficiency escape suppressed germanium detectors placed at 158° to the beam direction with four more at 134°. Five, lower efficiency (25 %) detectors were placed at both 79° and 101°. In a subsequent experiment, the thin target was replaced with a target of natural Ca of thickness of 630 μg/cm^2^ on a 20 mg/cm^2^ gold backing. The recoils stopped in the backing, thus providing high resolution data on γ−γ coincidences and angular correlations from which the decay scheme was constructed.

The AYEBALL experiment was followed up by a futher experimental study of ^62^Ga at the Niels Bohr Institute. Identification of transitions in 62Ga was achieved using the reaction 40Ca(^28^Si, αpn)^62^Ga, performed using an 88 MeV beam bombarding a 1 mg/cm^2^ self supporting target of enrichment 99.96 % in ^40^Ca. The γ-ray detection was afforded by the PEX spectrometer array [30], consisting of four, seven-element germanium cluster detectors [31] each with a BGO suppression shield. Two of the cluster detectors had their central crystals at an angle of 105° to the beam direction, with the other two being at 146°. Information on evaporated charged particles for channel identification was obtained using a 31-element silicon inner ball [32] surrounding the target position, in conjunction with an array of 15 BC501 liquid scintillator neutron detectors [33] positioned at forward angles.

**Fig. 3:**
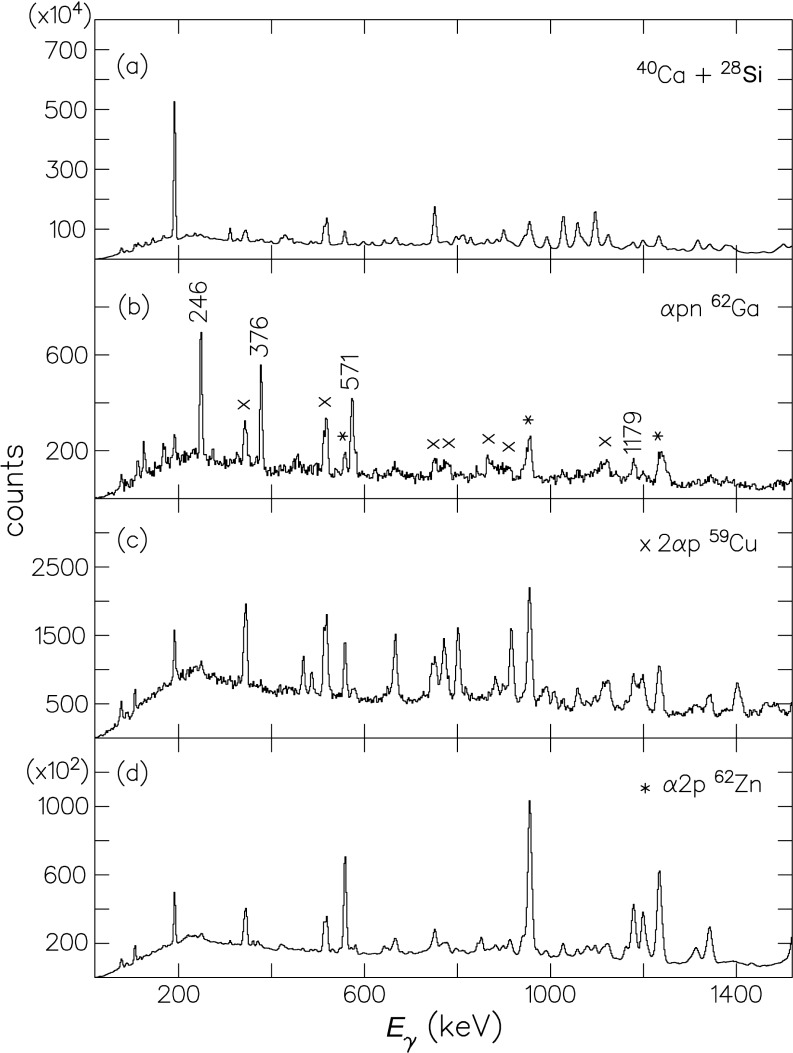
(a) Total projection of the γ−γ matrix with no channel condition, the dominant peaks are from the 3p evaporation channel ^65^Ga; (b) projection from the matrix selected by αpn showing the 246 keV, 376 keV, 571 keV, and 1179 keV ^62^Ga lines and contaminant lines from the α2p channel ^62^Zn (*) and 2αp channel ^59^Cu (*x*); (c) projection from the matrix gated by the 2αp channel; (d) projection from the matrix gated by the α2p channel.

Figure 3 a–d shows total projection spectra of the γ−γ coincidence matrices formed with several different channel selection criteria. The αpn channel leading to ^62^Ga is shown in Fig. 3b and clearly identifies the transitions at 246 keV, 376 keV, 571 keV, and 1179 keV, which we assign to ^62^Ga. Contaminant lines from the α2p channel (^62^Zn), and the 2αp channel (^59^Cu), which appear from a combination of the finite detection efficiency of the silicon ball and the misidentification of γ -ray events in the neutron detectors are also clearly marked. The transitions associated with the ^62^Ga gate are, in contrast, notably absent from the other two spectra.

**Fig. 4:**
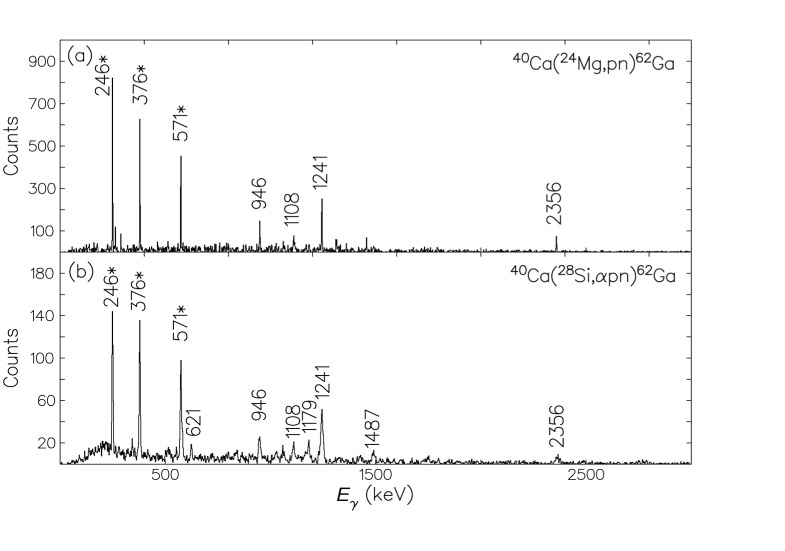
Transitions in coincidence with the 246 keV, 376 keV, and 571 keV rays in ^62^Ga (marked ^*^) from (a) the AYEBALL gold-backed target data and (b) the αpn-gated PEX data.

In the run at Argonne using the FMA, statistics were sufficient to associate γ rays with mass 62 but not good enough to unambiguously identify *Z* = 31 and hence the weak pn channel; accordingly, in the backed-target experiment, background-subtracted spectra were generated in coincidence with transitions identified using the PEX experiment. Figure 4 shows a comparison of the γ-ray spectra in coincidence with the 246 keV, 376 keV, and 571 keV transitions established in ^62^Ga from both the AYEBALL backed target data and the αpn gated PEX data.

**Fig. 5:**
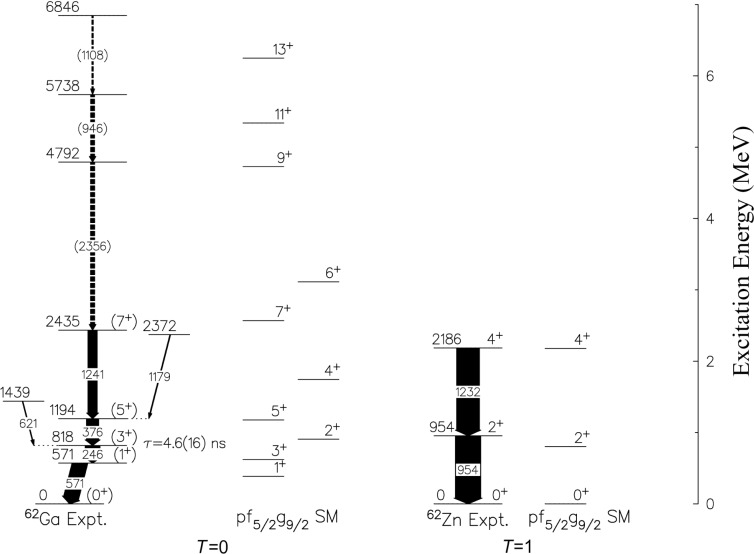
Decay scheme derived for ^62^Ga compared to the predictions of the *pf*_5/2_ *g*_9/2_ basis shell model. *T* = 0 states are shown on the left, and *T* = 1 states are on the right. The low lying levels in the *T_z_* = 1 isobar, ^62^Zn are included for comparison. Tentative experimental assignments are denoted by dashed lines and parentheses.

The decay scheme for ^62^Ga obtained in the current work is shown in Fig. 5. An isomeric state with a mean-lifetime of (4.6 ± 1.6) ns was identified at an excitation energy of 818 keV, from the decay of ^62^Ga reaction products which had implanted in the stopper foils placed in front of the silicon detectors in the charged particle detector. The lifetime was established using the recoil distance decay technique. Multipolarities for transitions identified in ^62^Ga were made using a combination of γ-ray anistropies from the PEX experiment and a DCO ratio analysis from the backed target AYEBALL data.

The observed levels in ^62^Ga and the lowest isobaric analogue states in ^62^Zn have been interpreted using a shell model analysis encompassing the *pf*_5/2_ *g*_9/2_ orbits with a 56Ni core. The results are shown in Fig. 5. The effective interaction is a realistic *G* matrix whose monopole part has been phenomenologically adjusted and which has been used previously in ^76^Ge and ^82^Se [34]. Calculations have been done with the shell-model code ANTOINE [35]. The difference between the *T* = 0 and *T* = 1 states is well reproduced as is the band structure on top of the 1^+^ state. Moreover, the calculated value of *B*(*E*2; 3^+^ → 1^+^) is 139 *e*^2^*fm*^4^ in excellent agreement with the value of 197(69) *e*^2^*fm*^4^ deduced from the measured mean life of the 818 keV level.

Clearly, the overall degree of collectivity is limited in ^62^Ga, and more information on non-yrast states would be necessary to distinguish the presence of any degree of coherent n-p pairing. However, while the full shell model calculation gives a very satisfactory result in this nucleus, such a method will become increasingly intractable as the number of valence nucleons, and hence collectivity, increases along the *N* = *Z* line. For this reason, a further calculation has been performed in the framework of the IBM-4.

## 5. Conclusions

**Fig. 6:**
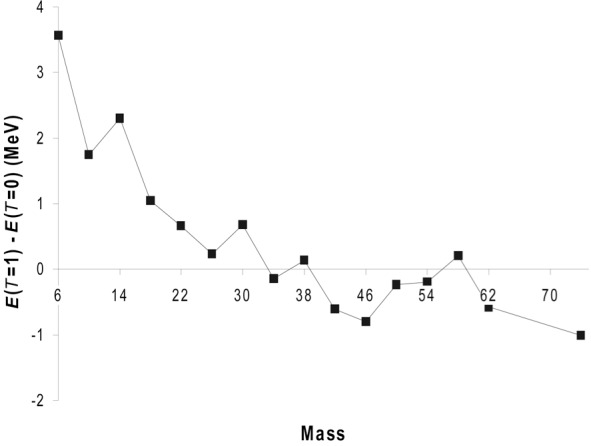
Difference in energy of the lowest *T* = 0 and 1 states versus mass.

A crucial feature of the new data presented above centres on the relative energy, of the *T* = 0 and *T* = 1 states in ^62^Ga. This can be viewed in the context of the overall systematics of Fig. 6 which show a gradual decrease in the separation energy *E*(*T* = 1)−*E*(*T* = 0) as mass increases along the *N* = Z line in this shell, this fall being accompanied by a gradual reduction in the contribution of *T* = 0 pairs. This behaviour is similar to that observed for the empirically derived interaction strength of the last neutron with the last proton and has been associated with the gradual erosion of SU(4) symmetry [19]. It seems likely that both features are linked to the change from *l*−*s* to *j*−*j* coupling as the strength of the spin-orbit interaction increases.

Clearly, more experimental information is needed, and the exploitation of radioactive beams will open up a whole new world in this context. Nevertheless, there are still many questions which can be answered by increases in sensitivity using stable beams. This is particularly true in the study of fusion evaporation reactions, where the enhancement in cross section offered by the use of exotic beams is offset by the reduction in beam intensity. There are, however, a host of properties which can *only* be studied with radioactive beams and where they offer unique information. Most importantly, the examples cited here show that information on the *non-yrast* states of highly exotic nuclei is required, which must come from the study of inverse reactions and *β*-decay, rather than fusion evaporation reactions.

Finally, there are even more intriguing possibilities which can be considered. One example is the study of the (n, γ) reaction with radioactive beams. The likely juxtaposition of such beams and neutron sources in the future, by virtue of the fact that both can use high-energy proton spallation as their production mechanism, suggests that such an idea is not entirely fanciful. Moreover, the large cross sections which characterise thermal neutron capture render such measurements, in principle, feasible.

## Figures and Tables

**Table 1:** Single particle orbits near the Fermi surface in ^74^Rb.

Nilsson orbit	$E_{q.p.}^{proton}(\mathrm{MeV})$	$E_{q.p.}^{neutron}(\mathrm{MeV})$
3/2^+^[431]	0.000	0.000
3/2^−^[312]	0.349	0.356
3/2^−^[301]	0.359	0.452
5/2^+^[422]	0.423	0.346
1/2^+^[440]	0.472	0.495
1/2^−^[310]	0.965	0.887
